# Metagenomics and Other Omics Approaches to Bacterial Communities and Antimicrobial Resistance Assessment in Aquacultures

**DOI:** 10.3390/antibiotics10070787

**Published:** 2021-06-28

**Authors:** Teresa Nogueira, Ana Botelho

**Affiliations:** 1 Laboratory of Bacteriology and Mycology, INIAV-National Institute for Agrarian and Veterinary Research, 2780-157 Oeiras, Portugal; ana.botelho@iniav.pt; 2 cE3c-Centre for Ecology, Evolution and Environmental Changes, Evolutionary Ecology of Microorganisms Group, Faculty of Sciences, University of Lisbon, 1749-016 Lisbon, Portugal

**Keywords:** aquaculture, bacterial communities, antibiotic resistance, microbial metagenomics

## Abstract

The shortage of wild fishery resources and the rising demand for human nutrition has driven a great expansion in aquaculture during the last decades in terms of production and economic value. As such, sustainable aquaculture production is one of the main priorities of the European Union’s 2030 agenda. However, the intensification of seafood farming has resulted in higher risks of disease outbreaks and in the increased use of antimicrobials to control them. The selective pressure exerted by these drugs provides the ideal conditions for the emergence of antimicrobial resistance hotspots in aquaculture facilities. Omics technology is an umbrella term for modern technologies such as genomics, metagenomics, transcriptomics, proteomics, culturomics, and metabolomics. These techniques have received increasing recognition because of their potential to unravel novel mechanisms in biological science. Metagenomics allows the study of genomes in microbial communities contained within a certain environment. The potential uses of metagenomics in aquaculture environments include the study of microbial diversity, microbial functions, and antibiotic resistance genes. A snapshot of these high throughput technologies applied to microbial diversity and antimicrobial resistance studies in aquacultures will be presented in this review.

## 1. Introduction

The need to provide adequate and safe food to a growing global population—9.8 billion people by 2050 [1], has intensified the importance of the aquaculture industry. Aquaculture is the fastest-growing animal food production sector worldwide and is becoming the main source of seafood for human consumption. The reduction in wild fishery stocks, a rising human population, a continuing demand for seafood and international trade has driven a great expansion of aquaculture during the last decades in terms of production and economic value. Aquaculture production provides almost half of the fish that are consumed worldwide, which has led producers to move towards intensive and semi-intensive production systems [2]. Nine of the top-ten ranked countries for aquaculture species diversity are in Asia, with China leading by a wide margin. The largest aquaculture producers outside Asia include Norway and Chile, which mainly produce Atlantic salmon (*Salmo salar*), and Egypt, which produces Nile tilapia (*Oreochromis niloticus*) [3].

The other side of this reality is that the use of intensive and semi-intensive practices for fish production leads to a higher concentration of animals in small spaces, substantially increasing the risk of contagious diseases [4]. Therefore, the prophylactic and therapeutic use of antimicrobials is currently employed to control disease outbreaks, and the substances widely used in aquaculture are the same as those licensed for therapy and the prophylaxis of infectious diseases in humans and livestock. Quinolones (i.e., oxalinic acid, flumequine, and enrofloxacin), tetracyclines (i.e., oxytetracycline), and phenicols (i.e., florfenicol) are the most widely antibiotics in aquaculture to control bacterial fish disease [5] and are administered mostly in food.

The use of antimicrobials in aquaculture, even in sub-inhibitory concentrations, may favor the emergence of durable and stable antimicrobial resistance (AMR) [6] and promote horizontal gene transfer (HGT) and mutagenesis in bacteria of the aquatic environment [7,8,9]. Due to the connections of epidemiological pathways between humans, animals, and the environment, the identification of the factors influencing AMR emergence and spread in animal production, such as aquacultures, will contribute to the ability to control resistance in the areas of food production, the environment, and public health [10].

Most studies have used culture-dependent methods to analyze antimicrobial resistant bacteria but it is known that, especially in complex matrices such as aquaculture environments and sediments, most of the bacterial population is non-cultivable and therefore culture-independent methods must be applied. High-throughput genomic technologies offer new approaches for environmental health monitoring, including the metagenomic surveillance of antibiotic resistance determinants (ARDs). This review focuses on the use of omics to appraise antibiotic resistance determinants in aquaculture.

Antibiotics are molecules, widespread in nature, which are naturally produced by bacteria and other microorganisms, as part of their natural life within a microbial community. In parallel, bacteria from microbial communities develops natural antibiotic resistance genes (ARG). Both antibiotics and ARG are as old as bacteria are [11,12,13]. For example, ancient DNA found in 30,000-year-old permafrost sediments has revealed a highly diverse collection of genes encoding antimicrobial resistance mechanisms to beta-lactam, tetracycline, and glycopeptide groups [12,14] that are associated with modern bacterial pathogens, highlighting the ancient origins of antibiotic resistance.

Antibiotic production and release into the environment can be a strategy that allows antibiotic-producing microorganisms to compete and communicate with each other within a microbiome (the collection of all the microorganisms and their genomes of that microbial community). Cooperation, competition, and inhibition among organisms within a microbiome are different aspects of the interplay between these different counterparts. Environmental antimicrobials are ubiquitous and naturally present in low concentrations, playing a very important ecological role in microbiome dynamics.

The resistome, as a collection of all the ARGs and their precursors in a microbial community, is a peculiarity of both pathogenic and non-pathogenic microorganisms since it is required for the survival and evolution of the bacteria in a dynamic environment [15,16]. The complex networks and interactions occurring between microbial species from diverse environments facilitate the gene flow, expanding the AMR between humans, animals, and the environment, resulting in a widespread issue.

Bacterial resistance to antibiotics relies on three main factors: the impermeability of the bacterial cell to the antibiotic molecule (e.g., the physicochemical properties of the molecules, the presence of efflux pumps etc.), the lack of the target molecules in the cell or the inactivation of the antibiotic compound by means of degrading enzymes [16]. Schmieder and Edwards reported at least four well-known mechanisms that contribute to antibiotic resistance in bacteria: “(i) the inactivation or modification of the antibiotic; (ii) an alteration in the target site of the antibiotic that reduces its binding capacity; (iii) the modification of metabolic pathways to circumvent the antibiotic effect; and (iv) the reduced intracellular antibiotic accumulation by decreasing the permeability and/or increasing the active efflux of the antibiotic.” [17].

Upon environmental changes, bacterial evolution relies on their genomic flexibility to adapt to the surrounding environment including, among others, the ability to protect themselves from toxic substances [18]. Moreover, the genetically determined resistance set up by given bacteria is efficiently transmitted to its clonal expansion and/or other bacterial species through mobile genetic elements, such as plasmids, transposons, and integrons [19]. The concerted activity of both genetic/heritable elements and phenotypic traits are involved in a wide array of metabolic functions, which have been considered essential for the onset and diffusion of the antimicrobial resistance.

Soils are inhabited by several different microbes creating a very rich reservoir of antibiotics and antimicrobial resistances, *Streptomyces* being one of the most important antibiotic-producing organisms in nature [20,21]. Likewise, Actinomycetes, a soil inhabitant, remains a very prolific source of novel antibiotics such as beta-lactams, tetracyclines, rifamycins, aminoglycosides, macrolides, and glycopeptides [21], which are all antimicrobials that have been widely used in medicine and agriculture for a long time.

In such a comprehensive approach, the antimicrobial use (and misuse) in the human, animal, and environmental settings, along with the global spread of the resistance mechanisms within and between these sectors are identified as the major AMR driving forces [22].

Currently, antimicrobials are being produced on a significant scale, are widely used in both human and veterinary medicine, livestock production and agriculture, and consequently are released in the environment in unnatural amounts, becoming an important and emerging contaminant [23]. Sewage waters from urban areas, hospitals, and animal farm effluents may be delivered into rivers and the environment, increasing the environmental contamination by antimicrobials, and unbalancing their natural concentration in ecosystems [24,25]. Consequently, both human and environmental bacteria can be under the selective pressure of different antibiotic concentrations and gradients. The evaluation of the impact of antibiotics usage in aquaculture and other animal productions on the environment and on the human/animal treatment efficacy can lead to a reduction in medication for animals. There is presently a tendency for a global policy to regulate the use of antibiotics, also as growth-promoting factors, worldwide.

Antimicrobial inhibitory concentrations can directly disrupt microbiome composition, by selecting resistant bacterial clones in natural biomes, whereas sub-inhibitory concentrations are important drivers for the evolution of antimicrobial resistance [26]. In particular, sub-inhibitory concentrations of fluoroquinolones, beta-lactams, and aminoglycosides can lead to a change in the microbiome dynamics, by triggering different genetic mechanisms of resistance such as: (i) HGT of antimicrobial resistance genes encoded in mobile genetic elements (MGEs), via the SOS system induction [26,27,28]; (ii) increasing genetic recombination; and (iii) increasing mutagenesis rate [29].

The molecular mechanisms of recombination, mutagenesis, and HGT by conjugation, transformation, and transduction may result in a loss of bacterial fitness [30,31]. These genetic events can also drive the emergence and spread of antimicrobial-resistance determinants encoded in mobile genetic elements (MGEs), such as some broad-host-range plasmids [15,26,32,33] that share the ability to remain in microbial communities. Thus, exposure to non-lethal doses of antimicrobials can prompt an adaptive response, by increasing genetic diversity and promoting the spread of resistance traits, particularly when the fitness cost is not negligible [34].

Therefore, one of the measures to control AMR is to preserve the efficacy of antimicrobial drugs used in human and veterinary clinical practice, animal production, agriculture, and aquaculture through the prudent use of antibiotics.

## 2. Microbial Communities and Antimicrobial Resistance in Aquacultures

In December 2017, the United Nations Environment Programme (UNEP) identified the environmental resistance to antibiotics as the major concern out of six emerging issues and referred to the fact that up to 75% of antibiotics used in aquaculture may be lost into the surrounding environment [35]. A strong positive correlation between flumequine and florfenicol consumption in aquafarms and the detection of their residues in surface water and sediment samples was identified [36], proving that the antibiotics used in aquaculture can reach the near aquatic environment. In Europe, Japan, and North America, the use of antibiotics in aquaculture is strictly limited to therapeutic applications where only a limited number of antibiotics are approved [37]. For example, between 1987 and 2013, the salmon production system in Norway reduced antibiotic use by 99%, and the use of vaccines, hygienic measures, and scientific research has been crucial for such an improvement [37].

Rearing animals in small tanks increases the stress of the animals and the incidence of infectious diseases, resulting in whole stock losses associated with economically important damages [4]. This leads to the use of antibiotics for both prophylactic and therapeutic purposes, promoting the selection and dissemination of antibiotic resistance genes by various routes (food, feed, and environment) [2,38,39]. Fish farming has been suggested as a reservoir of ARGs [40,41] and a significant correlation between the occurrence of ARGs and the concentration of antibiotics in aquaculture sites has been reported [42].

Today, there is no antimicrobial compound used exclusively in aquaculture. Thus, antibiotics intended for human and veterinary medicine sectors are also improperly used in the aquaculture which contributes to an exacerbation of the impact of AMR onset and dissemination [2,43]. Six antibiotic classes, listed by the World Health Organization (WHO), critically important in human medicine (aminoglycosides, macrolides, penicillins, quinolones, sulphonamides, and tetracyclines), have been widely used in both terrestrial and aquaculture husbandries, thus compromising their effective use in the treatment of infectious diseases in humans [2,44,45]. Moreover, the inadequate usage of antibiotics is associated with a reduced capability of the fish species to effectively metabolize the administered drugs. Therefore, antibiotic residues remain for long periods in fish meat, which promotes their entry into the food chain. Additionally, it is estimated that approximately 70 to 80% of antibiotic residues still active are eliminated in feces, contaminating wastewater and affecting the ecosystem [44].

Most of the aquaculture open farms use antibiotics and despite the need for a quarantine period, some antibiotic remnants may be discarded into the open sea [46]. Antibiotics are usually provided in feedstuffs [47] but not all the medicated feed is consumed by fish or crustaceans, and, after lixiviation, small amounts of antibiotics are released into the sediment or water and make contact with environmental bacteria that can develop resistance. Antibiotic resistance genes can be incorporated into aquaculture systems through manure, commonly used as natural fertilizer, containing bacteria with antibiotic resistances [48,49].

Currently, China has the world’s largest areas for marine farming production [50]; the industry of the Chinese mitten crab *Eriocheir sinensis* one of the main freshwater aquaculture industries in that country. However, the real situation of antibiotic resistance in aquaculture of *E. sinensis* is not yet adequately known [51]. Much attention has been paid to the distribution of antibiotics, antibiotic resistance genes (ARGs), and the bacterial composition of the community in this environment [52]. These include tetracycline and sulfonamides resistance genes, some of the emerging plasmid-mediated quinolone resistance (PMQR) genes, the new determinant of fosfomycin resistance, the widely disseminated emerging *floR* gene of human pathogens, and the chloramphenicol *catII*, *catB9* and *catB2* from aquatic *Photobacterium* sp., *Vibrio* sp. and *Shewanella* sp., respectively.

The use of antibiotics in aquaculture environments, combined with anthropogenic disturbances resulting from environmental contamination with antibiotics, not only affects bacteria in the microbiota of fish and shellfish, but also affects the aquaculture sediments. Marti and colleagues have compared the bacterial composition and the content of antibiotic resistant genes *bla*_TEM_, *ermB*, *qnrS* and *sul*I in two different ecosystems with high and low anthropogenic disturbance, respectively, and detected significant differences in bacterial community composition between the fish species, suggesting that anthropogenic activities promote the emergence and spread of antibiotic resistance in aquatic organisms [53].

The sediments below fish farms can be also enriched with ARG, as they are constantly receiving fish feces, such as those encoding resistance to sulfonamide (*sul1*), trimethoprim (*dfrA1*), tetracycline (*tet(32), tetM, tetO, tetW*), aminoglycoside (*aadA1, aadA2*), chloramphenicol (*catA1*), and efflux-pumps resistance genes (*emrB*, *matA, mefA, msrA*), together with class 1 integron-associated genes (*intI1*, *qacED1*), and transposases (*tnpA*) [54].

Oxytetracycline is a tetracycline broad-spectrum antibiotic that is widely used in aquaculture both for therapeutics and as a prophylactic agent. However, it has also been linked to the emergence of antibiotic-resistant bacteria in aquaculture environments, and to the contamination of fish meat and products with antibiotic residues and with human bacterial pathogens [55].

Many fish pathogens and aquatic bacteria, such as *Aeromonas salmonicida*, *A. hydrophila*, *Citrobacter freundii*, *Edwardsiella tarda*, *Yersinia ruckeri*, *Lactococcus garviae*, *Photobacterium damselae* subsp. piscicida, *Vibrio anguillarum*, *V. salmonicida*, *Photobacterium psychrophilum*, and *Pseudomonas fluorescens* [55], have been reported as having developed resistance to antibiotics as a consequence of antimicrobial exposure. Antibiotic resistance can be transferred from plasmids-encoding resistance genes from fish pathogens into other bacteria within the same genus but also to E. coli. That is the case of the multi-resistance plasmids harboring Salmonella Typhimurium DT104, such as tet(G) and flo-like genes, which confer resistance to tetracycline, florfenicol, and chloramphenicol that are present in the fish pathogenic bacteria *A. salmonicida*,* A. hydrophila*, *Edwardsiella tarda*, *Citrobacter freundii*, *Photobacterium damselae* subsp. piscicida, *V. anguillarum* and *V. salmonicida*, showing that some of the antimicrobial resistance factors in multiresistant *Salmonella* Typhimurium DT104, such as tet(G) and flo-like gene which confer resistance to both florfenicol and chloramphenicol, are also present in some fish pathogenic bacteria [55]. These observations reinforce the idea that aquaculture environments can act as reservoirs of antibiotic resistance genes.

*Vibrio parahaemolyticus* and *Vibrio vulnificus* are the leading foodborne bacterial pathogens that cause seafood associated infections and death in the US. They are reported as being usually susceptible to most antimicrobials of veterinary and human significance; however, Elmahdi and colleagues [56] have performed a comparative study across many different countries worldwide and have concluded that of the antibiotic resistance profiles involved, regardless of the country, ampicillin, penicillin, and tetracycline were frequently observed in aquacultures. The presence of multiple-antibiotic resistant bacteria in seafood and aquatic environments is a major concern in fish and shellfish farming and human health.

In a comprehensive metagenomic study on the structure of bacterial communities, and the abundance and diversity of ARGs, as well as MGEs in the three Chinese mitten crab aquaculture ponds in Jiangsu Province, China contained in [51] it was revealed that resistance to bacitracin was very prevalent in the water, while sediments were enriched in multidrug resistance traits. There was also a significant correlation between MGEs (particularly plasmids) and ARGs which may cause a potential risk to human health [51].

Another work using quantitative PCR and bacterial culture-dependent methods to evaluate ARGs and antibiotic resistant bacteria in marine fish farming areas indicated that the *sul* and *tet* family genes were widely distributed in Hainan, China; specifically, *sul1* and *tetB* were the most abundant genes detected [57]. The most prevalent species found belonged to the genera *Vibrio*, *Acinetobacter*, *Pseudoalteromonas*, and *Alteromonas* which are opportunistic pathogens with a high resistance rate to oxytetracycline. It was found that salinity also has an important effect on the abundance of ARGs and antibiotic resistant bacteria in the marine fish farming area [57,58]. On the southeast coast of China it has been shown that *flo*R, *sul*II, *sul*I, *str*B, *str*A, *aad*A, and *tet*S were the prominent ARGs with high detection frequencies ranging from 30.9 to 51.1% in total samples. This study also showed that ARGs are more abundant in freshwater aquatic animals than in marine animals, reflecting a discrepancy in the cultivation patterns between the freshwater and marine aquacultures [57].

In the Drwęca river in Poland, fish farming drove an increase in the diversity of tetracycline-resistance genes [59]. Resistance to tetracyclines (tetracycline, oxytetracycline or chlortetracycline) are among the most frequently detected in aquacultures across the world [60] and can have an impact on the water quality. The Polish Drwęca River study showed that fish farming influenced the quality of the water directly by increasing the diversity of tetracycline-resistance genes by HGT induced between *Aeromonas* sp. and *Acinetobacter* sp. into *E. coli* [59]. Therefore, we can conclude that the anthropogenic activities and the pollution of aquatic environments, particularly in relation to antibiotic residuals, can trigger the emergence and spread of antibiotic resistance [53].

Aquaculture is a new food production sector that has been increasing dramatically over the last two decades due to the high demand for a healthy protein source [61]. The aquaculture production figures indicate a substantial increase in the relative contribution of aquaculture to total fish consumption from 5% in 1962 to 49% in 2002 [62]. In 2014, food fish produced via aquaculture systems reached 70.5 million tons [60], and in 2018 seafood provided almost 20% of all animal protein in diets, globally [63].

Most aquaculture production units (>90%) are located in Southeast Asian countries, where fish farming is practiced in oceanic networks or contained in lagoons or reservoirs, and often integrates fish farming practices that use waste from animal husbandry potentially contaminated with antibiotic residues [64]. Overcrowding, unhygienic measures, and other manipulations may promote the spreading of bacterial infection and concomitantly an increase in antimicrobial use particularly in the shrimp and salmon industries [65].

Although disease control in fish farming can be vaccine-based, antimicrobial treatment in medicated feed or bathing with fluoroquinolones, florfenicol, tetracycline, sulfonamides, and amoxicillin are often used. Antimicrobial substances are thus used in fish farming industry for therapeutic and prophylactic purposes and in some countries, albeit not in European ones, they are also added to animal feeds as growth promoters [41]. Therefore, the trend towards antimicrobial and multidrug resistance in aquaculture is increasing dramatically and is of major concern [61,66]. In fact, the antimicrobial agents that are most widely used in aquaculture are the same as those authorized for therapy and the prophylaxis of infectious diseases in humans and animals [67,68]. Fish are also reservoirs of zoonotic pathogens that can be transmitted to humans in aquaculture facilities or through food [60,69]. The OIE have conveyed that tetracyclines were the most commonly reported antimicrobial class among the 116 countries providing quantitative data, accounting for 34.5% of those used from 2015 to 2017 [70].

The widespread application of antimicrobials to fish will lead to the release of uneaten feed and fecal particles dispersed in water, which may contain residues that persist in the surrounding environment. In addition, as fish do not metabolize antibiotics effectively, 70% to 80% of the active substance can pass into the environment through the feces or remain in the fish tissues for long periods of time [2]. It is known that bacterial populations exposed to low antimicrobial levels concentrations are selected for AMR [61,65,66]. In aquaculture, AMR can develop in the gut microbiota of fish and on other bacteria in the aquatic environment as a result of antimicrobial pressure. Determinants of antimicrobials resistance have been detected in aquatic bacteria, some of which may be pathogenic to humans [2,7,71].

In a bibliographic analysis to review the use of antibiotics in the top 15 aquaculture producing countries between 2008 and 2018 it was pointed out that 67 antibiotic compounds were used in 11 of the 15 countries, including oxytetracycline, sulphadiazine, and florfenicol, and that on average, countries used 15 antibiotics; Vietnam, China and Bangladesh were the top users [72].

The emergence of β-lactamase- and carbapenemase-producing Enterobacteriaceae at integrated fish farms is particularly worrisome [73]; carbapenemase-producing *E. coli* from oysters, aquatic shrimps, and lakes has been reported in Brazil [66]. In addition, the detection of carbapenemase in non-fermentative Gram-negative organisms, such as *Stenotrophomonas*, *Myroides,* and *Pseudomonas* spp. isolates, recovered from frozen seafood imported from Asian countries, suggests that non-pathogenic bacteria excluded from antimicrobial surveillance programs may act as reservoirs of the carbapenemases genes in the food supply [74]. Recently, researchers are questioning whether fish commonly used in raw preparations such as sushi and sashimi constitute a public health problem [75].

Resistance to more than one antibiotic has also been described in *Vibrio parahaemolyticus, Clostridium difficile*, and some *Enterobacteriaceae*, among others, isolated from bivalve mollusks from coastal areas [76,77,78]. These food products pose a potential risk to humans as they are consumed without adequate heat treatment or even raw, such as flat oysters (*Ostrea edulis*) and large scallops (*Pecten maximus*). Bivalve play an important role in resistance transfer determinants, as they are suspension feeders, actively filtering, retaining, and concentrating particles, including free-living or particulate-bound bacteria, from the surrounding water [76]. Thus, the presence of antibiotic-resistant bacteria in fish, seafood, and mollusks may represent a threat to human health, and could also result in the transfer of resistant determinants to other clinically important bacteria [41].

## 3. Omics Technologies to Address Microbial Communities

The conventional methods for the detection of antibiotic resistance are based on growth inhibition assays in broth or agar disc diffusion, in which the minimal inhibitory concentration (MIC) of particular antibiotics is estimated for each bacterial isolate. With this procedure only a few bacterial isolates can be studied at a time, contrasting with the millions of bacterial species that can be present within aquaculture facilities and in the effluent-receiving ecosystems [79]. Another problem, associated with culture-based systems, is the culturing time, which may take from 1 to 2 days for fast-growing bacteria to several weeks for slow-growing species.

Methods involving quantitative PCR and microarray technologies have been developed, but they only detect the presence of specific well-studied genes related to antibiotic resistance [80]. They cannot be used for wide-spectrum screening, and thus, the real potential of non-culturable species as antibiotic resistance reservoirs is ignored. The outbreak of next-generation sequencing circumvents these limitations because it is a culture-/amplification independent technique. Therefore, these technologies allow for a deeper insight into the genomic information of most bacteria, leading to the detection of novel resistance genes [81]. The metagenomic (beyond the single genome study; *meta*- meaning transcendent in Greek) approach provides information regarding the presence, absence, or modification of the genes responsible for antibiotic resistance and, furthermore, the discovery of novel genes is faster.

Metagenomics has emerged in the last decade as a promising centerpiece that attempts to analyse the multiple genomes contained within a microbial niche or biome [82]. Thus, instead of collecting live microorganisms from a microbial community to be cultured or observed in the laboratory, the isolation of DNA directly from a sample can provide information related to the diversity of the microorganisms thriving in certain areas and can inclusively reveal information related to their functions and biological roles. Until recently, the classic bacteriological culture was the standard procedure to study bacterial communities, but with the advent of genomic approaches, namely metagenomics, a new window to study the world of microorganisms has opened.

The term microbiome refers to any microbial community inhabiting a biome, together with the set of their genomes. It can be composed from a multitude of different types of microorganisms, 99.99% of which are estimated to remain unknown [83] or unculturable [84]. The entire microbial community behaves as a biological system, as the group of organisms responds collectively and adapts to the environmental changes. Individual bacterial cells from a consortium can perform metabolic functions in a cooperative way [85,86], providing group advantage and indirect benefit to all species involved [87], which is crucial for understanding microbial dynamics and environmental adaptation. The term metagenomics thus refers to the analysis of all genomes from all microorganisms present in a sample, even those difficult to culture by classic bacteriological methods.

High throughput screening technologies help to obtain the holistic view of different levels of biological molecules: genes (genomics), genetics (metagenetics), mRNA (transcriptomics), proteins (proteomics), lipids (lipidomics), and metabolites (metabolomics) that make up and sustain the life of all cells [88,89,90]. The main reason behind omics technologies growing popularity in biological research is their ability to provide an understandable view of a complex biological system as a whole. Genetic information stored in the genome translates into its phenome (set of all phenotypes being expressed), performing appropriate biological functions primarily through protein.

Since 1977, 16S ribosomal RNA coding sequences (16S rRNA) have been used for bacterial phylogenetic analysis purposes [91], as they evolve at a very slow rate. The 16S rRNA study from various environments has provided strong evidence for the existence of uncultured microorganisms and an almost complete picture of the real microbiome. Currently, other sequence analysis algorithms are being used to reconstruct the evolutionary histories of organisms (phylogenomics) and classify microorganisms [92].

Metagenomics deals with a microbial community or microbiome as a whole and allows not only for the evolutionary dynamics of a microbiome to be addressed, but also for comparative studies of different microbiomes to be carried out. Metagenomic sequencing reveals microbial identities and functional gene information, including DNA from microbes with vastly varying physiological states. Therefore, metagenomics enables the prediction of the community functional potential.

The next and most recent concept, termed the metaphenome, refers to the set of all the expressed phenotypes encoded in the metagenome and in the environment [93]. Metaphenomics is the understanding of the metaphenome, the product of the combined genetic potential of the microbiome and the available resources (biotic and abiotic constrains present in the environment).

By sequencing DNA directly from microbial community samples without previous culture and isolation steps, many different individual bacterial genomes, all mixed into one sample are brought together with the hope of capturing all the diversity of organisms and functions present. The main asset of a metagenome is to represent the community of microorganisms living in a particular environment or biome. Metagenomic analysis allows the phylogenetic diversity (every individual bacterium) in the community to be evaluated by performing an alpha diversity analysis. To obtain species or taxa diversity, we can try to answer three different and complementary questions: (i) how many different species or taxa can be detected in a microbiome? (ii) how are microbe diversities balanced with each other? (iii) do we have species uniformity (similar abundance level) or do some species dominate over others? [94].

**Figure 1:**
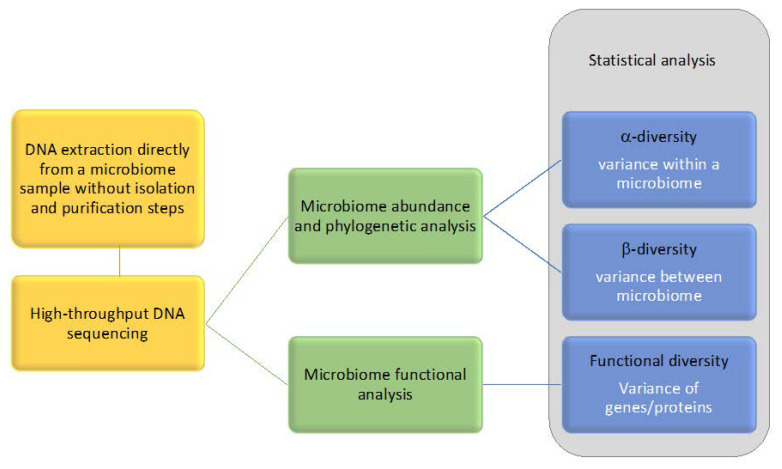
Main steps of metagenomic analysis.

During a metagenomic analysis, the following steps should be performed: (i) extraction of microbial DNA directly from a biological sample; (ii) followed by high throughput sequencing; (iii) sequential processing of bioinformatics; (iv) and statistical analysis [95]. Figure 1 outlines the main steps of the metagenomic analysis. Although laboratorial processing steps can be reduced in metagenomics studies, some powerful bioinformatic tools and processing skills are required to generate biological knowledge [95]. As in silico approaches are gaining ground over the wet lab component of microbiology, several new bioinformatic pipelines and platforms have been designed to identify and compare bacteria, allowing 16S sequence data derived from metagenomics to be processed. Some examples are: CopyRighter [96], Dada2 [97], Deblur [98], Greengenes [99], MOTHUR [100], MicroPro [101], PAPRICA [102], PhylOTU [103], PICRUSt2 [104], QIIME2 [105], RDP16 [106], rrnDB [107], SILVA [108], Tax4Fun [109], UPARSE [110] and VITCOMIC2 [111] (Table S1, See supplementary material).

The most striking feature of metagenomic analysis is that it deals with sequences issuing from both known and unknown microorganisms belonging to the same microbial community. Metagenomic analysis also allows the distribution of these different Operational Taxonomic Units (OTUs) to be evaluated as an estimated measure of their relative abundance (the “how many?” question). The Shannon index measures the diversity of a microbiome allowing whether there are bacterial taxa that are dominant over others or, on contrary they are balanced to each other to be ascertained.

The evaluation of the 16S gene copy number also allows data to be normalized, so that different microbiomes can be compared. This information is of great value as it underlies the analysis of microbial diversity. This normalization enables comparative analysis within different environments (beta diversity) to be performed and makes it possible to establish how different the microbial composition is in one environment compared to another.

Metagenomics has progressively replaced molecular techniques based on PCR (Polymerase Chain Reaction) amplification steps, such as cloned ribosomal amplicon sequencing libraries, as these PCR-based approaches were limited to bacteria and did not provide information on the metabolic capabilities of the studied microbiomes. High throughput metagenomic technologies produce a large amount of data, including a great number of genes, gene expression/protein abundance, in a single method or a combination of different methods.

Another promising aspect of metagenomics is thus the ability to allow the repertoire of bacterial genomic traits of a microbial community to be characterized collectively, that is, for functional annotations of all genomic sequences in the community to be performed. To accomplish this task, sequence readings issued by post-Sanger throughput sequencing methods must be assembled, followed by gene prediction and annotation so that the functional diversity can be addressed. A functional metagenomics analysis provides information on coding sequences for any trait, namely virulent factors, or antimicrobial resistance determinants [112,113]. Novel genes and gene products can be also discovered by metagenomics include several hydrolytic enzymes, novel molecules, and antimicrobial compounds.

Some available tools designed specifically for genome annotation are: the Integrated Microbial Genome Expert Review (IMG/ER) system from the Joint Genome Institute; the National Microbial Pathogen Data Resource’s RAST (Rapid Annotation using Subsystems Technology) server; JCVI (J. Craig Venter Institute) annotation service; and the University of Maryland’s IGS (Institute for Genome Sciences) annotation engine [114]. The combination of a phylogenetic profiling approach and a functional analysis can provide answers to the following questions: “who is there?” and “what are they doing or can they do?” [115]

## 4. Omics Technologies to Characterize Aquatic Resistomes

The more conventional target sequence polymerase chain reaction (PCR)-based amplification methods are not suitable to identify and quantify ARGs in microbial communities. During the last decades, the development of high-throughput sequencing technologies have enabled a metagenomic analysis to be performed to study the ARGs in diverse ecological environments [116]. For example, using a metagenomic approach, Escudeiro et al. [112] showed that there is a co-selection of resistance and virulence determinants in bacterial communities, particularly in human gut microbiomes.

Many different antibiotic resistance gene databases have been generated recently with the aim to help in finding and annotating ARGs. For a review on existing antibiotic resistance gene data resources see [117] and Table 1 lists of some of the most recently used such as, for example Resfams, which is a curated database of protein families organized by ontology [118], Structured Antibiotic Resistance Genes (the SARG) with a hierarchical structure (ARGs type-subtype-reference sequence) ARGs-OAP [119], Comprehensive Antibiotic Resistance Database (CARD 2020) [120], or Antibiotic Resistance Gene-ANNOTation (ARG-ANNOT) [121] that is restricted to experimentally confirmed proteins conferring antibiotics.

Culture-based methods are not suitable for comprehensive studies on the diversity and abundance of ARGs, nor for the occurrence of mobile genetic elements (MGE). It is essential to establish the ideal methodology for quantitatively and accurately assessing the antimicrobial sensitivity of environmental microbes. Therefore, studies of antibiotic resistome using metagenomic approaches in aquaculture environments have also been developed [125].

High throughput methods increase the processing, data production, and analytic capabilities of these techniques. However, as the cost of next-generation sequencing decreases, more up-to-date approaches, such as metagenomic analysis, have been used to analyse the resistome and the mobilome of different matrices, namely toilet waste [126]. This technology has enabled the characterization of bacterial communities and the analysis of different characteristics such as ARG and MGE. As a result of HGT, mobile genetic entities that constitute mobilome can be incorporated into the pangenome of terrestrial bacteria, including human pathogens, by binding aquatic and terrestrial resistomes and hindering the treatment of human infections [127,128].

In China, several studies have focused on antibiotics and ARGs in the aquatic environment because of the increased consumption of antibiotics in this country [52,129,130]. Recent studies relying on metagenomics showed remarkable differences through the year, and from one aquatic environment to another [131,132], on the antimicrobial resistance profiles and on the relative abundance of major bacterial phyla [133].

Hospital and municipal wastewater were found to have a higher diversity and mean abundance of ARGs compared to treated wastewater and effluent and surface water [134]. This is consistent with Fitzpatrick and Walsh’s in silico search for ARGs from different ecological niches [116]. High throughput sequencing-based metagenomic approaches have been used to comprehensively investigate the structure of bacterial communities, the abundance and diversity of ARGs, as well as MGEs. Untreated sewage metagenomic analysis was used to characterize bacterial resistomes from 79 sites in 60 countries [135]. Systematic differences in the abundance and diversity of ARGs were found between Europe/North America/Oceania and Africa/Asia/South America. AMR gene abundance strongly correlates with socioeconomic, health and environmental factors, which were used to predict the abundance of ARGs in all countries of the world. The diversity and abundance of those genes varies by region, and improved sanitation and health can potentially limit the overall burden of AMR. Sewage metagenomic analysis is an ethically acceptable and economically viable approach to the continuous global surveillance and prediction of AMR.

In a metagenomic study performed with samples issuing from the highly human-impacted catchment of the Beijiang River and its source in China revealed differences in the ARG profiles (both at the level of the diversity and abundance), in the bacterial community and in MGE elements, in surface waters compared with sediments [136]. These metagenomic analyses enabled the dissemination of antibiotic resistance in two different aquatic matrices to be addressed: superficial waters compared to the more structured sediments. Multidrug and bacitracin resistance genes were the most predominant ARG types, and the most shared ARGs were those conferring resistance to the clinically relevant antibiotics. These results strengthen the belief in the pivotal importance of MGEs in sharing resistance between humans, animals, and the environment, and thus their risks to public health [136].

A similar study was performed using samples from the gut and related aquaculture environments (water and sediment) of shrimp. The authors found dozens ARGs belonging to 13 to 15 different types in the shrimp gut, pond water and sediment samples, and evidence that MGEs contributed to 74.46% of the resistome variation associated with the presence of *Aeromonas*, *Yersinia*, and *Clostridium* species [137]. Efflux pump and target modification were the predominant resistance mechanisms found in this study.

The drawback of the metagenomic techniques is the loss of the approach of the individual organism or bacterial clone. Returning to wet lab microbiology approaches would include the isolation and cultivation of all possible species present to infer the composition of the bacterial pool. However, despite the advantages of culture-dependent techniques, namely its low cost and the collection of biological material, such as isolates, which enables further in-depth studies, this approach provides a highly restricted view of the microbial community.

On the other hand, culturomics has developed culture methods to identify unknown bacteria as part of the revival of culture techniques. As it combines various culture conditions and methods for the rapid identification of bacteria, the culturomics approach has allowed the cultivation of hundreds of new microorganisms with diverse and specific features. Culturomics may be defined—by an analogy with metagenomics—as an approach allowing an extensive assessment of the microbial composition by high-throughput culture. While metagenomics only provides sequencing data, with culturomics it is possible to directly test the strains originating from the microbiota analysed. Culturomics can be further developed thanks to automation, miniaturization, and other improved technologies.

This new approach has been applied to the study of complex microbial communities, such as the human gut, and may also be involved in antibiotic resistance research [138]. It has the potential to detect minority populations, is not restricted to Eubacteria, allows the identification of new species, and the study of interactions between different bacterial strains present in a given microbiota. Another advantage of using culture instead of molecular approaches is the additional information on the viability of detected microorganisms. Lagier and colleagues have identified as many as 32,500 different colonies recovered from three human stools [139] using matrix-assisted laser desorption ionization time-of-flight mass spectrometry (MALDITOF-MS) which not only represents a revolution in clinical diagnostic laboratories [140,141], but also represents a revolution in microbial ecology. It can also be coupled with intelligent incubators and an automated colony collection system. As a proof of concept, the authors applied 212 different culture conditions to grow 340 bacterial species and 5 different fungi, as well as the largest virus ever found in a human sample [139]. In addition, they discovered 32 new species, corresponding to about a third of those that were identified through culture, from samples of human intestines, for a decade. The authors listed the highly innovative culture conditions and selective approaches that were applied to circumvent the high growth rate of Enterobacteriaceae, such as: (i) removal of *E. coli* by specific lytic phages; (ii) various antibiotic cocktails; (iii) heat destruction of non-sporulated bacteria; (iv) different selective media; and (v) amoebal co-culture [139]. In addition, various atmospheres and incubation temperatures were used, as well as a large variety of enrichment culture media as similar as possible to that found in the human gut.

A parallel metagenomic analysis of the same samples identified a total of 638 phylotypes, including 282 known bacterial species [139]. Curiously, only 51 species identified by 16S rRNA sequencing were among the 340 cultured species. Other results obtained from culturomics showed that culturomics and metagenomics are complementary techniques for the identification of species, since the studies developed by Bilen and collaborators revealed that only about 15% of the bacteria were found simultaneously by the two methods [142]. Furthermore, the availability of cultures enables genome sequencing and many possible biotechnological applications [143]. The combination of both culturomics and metagenomics approaches will significantly advance the understanding of the role of microbes and their specific properties.

The current methodologies based on omics methods will also improve resistome assessment in the environment.

## 5. Concluding Remarks

Due to the increase in anthropogenic activities, antibiotics are now an important contaminant of the habitat, namely the aquatic and aquaculture environments, and thus can potentially enter the human food chain. Some of these antibiotic residues may be of critical importance to human health and, therefore, bacteria that share resistance to these antibiotics should be kept under epidemiological control.

Recently, metagenomics has become not only a promising tool for studying microbial diversity in aquatic environments, but also has become a useful way to evaluate the diversity of antimicrobial resistance characteristics present in a microbiome. On the other hand, culturomics, a technique that uses high-throughput bacterial culture methods with automation equipment, is reviving some traditional trends in microbiology.

In conclusion, we would like to emphasize that large-scale analysis is a very informative approach when addressing unstructured aquatic environments and microbial communities, and its use should be widespread.

## Figures and Tables

**Table 1:** Bioinformatics databases of antibiotic resistance genes.

Name of the Tool	Ref.	Link (Accessed on 24 June 2021)
AMRFinderPlus	[122]	https://github.com/ncbi/amr/wiki
ARG-ANNOT	[121]	https://github.com/tseemann/abricate/pull/82
ARGs-OAP	[119]	https://galaxyproject.org/use/args-oap/
CARD 2020	[120]	https://card.mcmaster.ca
MUSTARD	[123]	http://mgps.eu/Mustard/
Resfams	[118]	https://github.com/dantaslab/resfams
ResFinder 4.0	[124]	https://cge.cbs.dtu.dk/services/ResFinder/

## Supplementary Materials

The following are available online at https://www.mdpi.com/article/10.3390/antibiotics10070787/s1. Table S1: Bioinformatics tools for rRNA quantifications and analysis. References [95,96,97,98,99,101,102,103,104,105,107,108,109,110,111,144,145,146,147,148,149,150,151,152,153,154,155,156] are cited in supplementary material file.
