# Supplementary material for: Metagenomics and Other Omics Approaches to Bacterial Communities and Antimicrobial Resistance Assessment in Aquacultures
Source: Antibiotics (Basel). 2021 Jun 28;10(7):787. doi: 10.3390/antibiotics10070787 (PMC8300701; doi:10.3390/antibiotics10070787)
Supplement: Supplementary file 1 [file antibiotics-10-00787-s001.zip › antibiotics-1225118 Supplementary File.pdf]

Supplementary material

Bioinformatics tools to analyse metagenomic taxonomy

As *in silico* approaches are gaining ground over the wet lab component of microbiology, several new bioinformatic pipelines and platforms have been designed to identify and compare bacteria, allowing 16S sequence data derived from metagenomics to be processed, and are listed in the Table.

Table S1. Bioinformatics tools for rRNA quantifications and analysis.

**Name**	**Link** (accessed on 24 June 2021)	**Reference**
CopyRighter	https://github.com/fangly/AmpliCopyrighter/releases	[96]
Dada2	https://benjjneb.github.io/dada2/	[97]
Deblur	https://github.com/biocore/deblur	[98]
Greengenes	http://greengenes.lbl.gov	[99,144]
MicroPro	https://github.com/zifanzhu/MicroPro	[101]
MOTHUR	http://mothur.org	[145]
PAPRICA	https://www.polarmicrobes.org/introducing-paprica/	[102]
PhylOTU	https://github.com/sharpton/PhylOTU	[103]
PICRUSt	https://github.com/picrust/picrust	[146]
PICRUSt2	http://picrust.github.io/picrust/	[104]
QIIME 2	http://qiime.org	[105]
RDP16	http://rdp.cme.msu.edu/	[95,147,148]
rrnDB	https://rrndb.umms.med.umich.edu	[107]
SILVA	https://www.arb-silva.de	[108,149]
Tax4Fun	http://tax4fun.gobics.de	[109]
UPARSE	https://drive5.com/uparse/	[110]
VITCOMIC2	http://vitcomic.org	[111]

MOTHUR [145] and QIIME (Quantitative Insights Into Microbial Ecology) [108], together with QIIME 2 [105], are the most commonly used software [149], MOTHUR being the most cited bioinformatics tool for analysing 16S rRNA gene sequences [150]. They are both user-friendly and produce similar results. DADA 2[97], Deblur [98] does also offer similar results.

The assignment of taxonomy depends on the comparisons between the consensus sequences of OTU with the known microbial 16S rRNAs. Some microbial 16S rRNA reference databases are now available for taxonomic classification or binning, such as SILVA [108,149], Ribosomal Database Project (RDP) [95,147,148], Greengenes [101,144], NCBI [153,154] as examples. SILVA is the largest, while Greengenes is the smallest of all of the above [155]. The OTU count provides information on the number of distinguishable taxa in each sample (the questions “who's there?” or “how different?”). Thus, it allows one to estimate the microbial diversity or richness present in a metagenome [154]. VITCOMIC2 (Visualization tool for Taxonomic COmpositions of Microbial Community) is software designed for visualizing the phylogenetic composition of microbial communities based on 16S rRNA gene amplicons and shotgun metagenomic sequencing [111]. PhylOTU is a high throughput procedure that quantifies microbial community diversity and solves new taxa from metagenomics into OTUs [103].

rrnDB a searchable database that records variations in ribosomal RNA (rrn) operons in microorganisms of Bacteria and Archaea Domains, and it also allows the abundance of each species or taxa present in a microbiome to be quantified. It records the information on the 16S gene copy number of microorganisms. It is a searchable database documenting the variation in ribosomal RNA (rrn) operons in Bacteria and Archaea microorganisms [107,156], as it is linked to the NCBI and RDP taxonomy databases.

UPARSE [110] software is highly accurate, delivering results in far fewer operational taxonomic units (OTUs). The bioinformatics pipeline consists of five main steps: pre-processing and quality control filtering, OTU binning, taxonomy assignment, abundance table construction, and phylogenetic analysis [95].

The software PICRUSt [146] and PICRUSt2 [104] (Phylogenetic Investigation of Communities by Reconstruction of Unobserved States) allows the functional composition and abundance of a metagenome to be predicted using 16S rRNA and other marker gene sequencing data and a reference genome database. From OTU data, it is possible to accurately predict the abundance of gene families in environmental and host-associated communities, with quantifiable uncertainty [104]. CopyRighter is another useful tool designed to estimate the diversity of microbial abundance. It improves the accuracy of microbial community profiles by correcting the copy number of lineage-specific genes [96]. PAPRICA (PAthway PRediction by phylogenetIC plAcement) uses phylogenetic placement to conduct the metabolic inference-based approach, allowing precise inferences for overrepresented taxa and allowing for increased inference accuracy for taxa that have many sequenced genomes [102]. Tax4Fun is an open-source R package that provides for the functional capabilities of microbial communities based on 16S rRNA datasets. It provides a good approximation to the functional profiles obtained from shotgun metagenomic sequencing approaches [109].

The most striking feature of metagenomic analysis is that it deals with sequences issuing from both known and unknown microorganisms belonging to the same microbial community. MicroPro [101] is a software that is aimed at profiling organisms with greater precision, that takes advantage of unknown sequences.

References

95. Calle, M.L. Statistical Analysis of Metagenomics Data. *Genom. Inf.* **2019**, *17*, e6, doi:10.5808/GI.2019.17.1.e6.
96. Angly, F.E.; Dennis, P.G.; Skarshewski, A.; Vanwonterghem, I.; Hugenholtz, P.; Tyson, G.W. CopyRighter: A Rapid Tool for Improving the Accuracy of Microbial Community Profiles through Lineage-Specific Gene Copy Number Correction. *Microbiome* **2014**, *2*, 11, doi:10.1186/2049-2618-2-11.
97 Callahan, B.J.; McMurdie, P.J.; Rosen, M.J.; Han, A.W.; Johnson, A.J.A.; Holmes, S.P. DADA2: High-Resolution Sample Inference from Illumina Amplicon Data. *Nature Methods* **2016**, *13*, 581–583, doi:10.1038/nmeth.3869.
98 Amir, A.; McDonald, D.; Navas-Molina, J.A.; Kopylova, E.; Morton, J.T.; Zech Xu, Z.; Kightley, E.P.; Thompson, L.R.; Hyde, E.R.; Gonzalez, A.; et al. Deblur Rapidly Resolves Single-Nucleotide Community Sequence Patterns. *mSystems* **2017**, *2*, e00191-16, /msys/2/2/e00191-16.atom, doi:10.1128/mSystems.00191-16.
99 DeSantis, T.Z.; Hugenholtz, P.; Larsen, N.; Rojas, M.; Brodie, E.L.; Keller, K.; Huber, T.; Dalevi, D.; Hu, P.; Andersen, G.L. Greengenes, a Chimera-Checked 16S RRNA Gene Database and Workbench Compatible with ARB. *Appl. Environ. Microbiol.* **2006**, *72*, 5069–5072, doi:10.1128/AEM.03006-05.
101 Zhu, Z.; Ren, J.; Michail, S.; Sun, F. MicroPro: Using Metagenomic Unmapped Reads to Provide Insights into Human Microbiota and Disease Associations. *Genome Biology* **2019**, *20*, 154, doi:10.1186/s13059-019-1773-5.
102 Bowman, J.S.; Ducklow, H.W. Microbial Communities Can Be Described by Metabolic Structure: A General Framework and Application to a Seasonally Variable, Depth-Stratified Microbial Community from the Coastal West Antarctic Peninsula. *PLOS ONE* **2015**, *10*, e0135868, doi:10.1371/journal.pone.0135868.
103 Sharpton, T.J.; Riesenfeld, S.J.; Kembel, S.W.; Ladau, J.; O'Dwyer, J.P.; Green, J.L.; Eisen, J.A.; Pollard, K.S. PhylOTU: A High-Throughput Procedure Quantifies Microbial Community Diversity and Resolves Novel Taxa from Metagenomic Data. *PLoS Comput. Biol.* **2011**, *7*, e1001061, doi:10.1371/journal.pcbi.1001061.
104. Douglas, G.M.; Maffei, V.J.; Zaneveld, J.; Yurgel, S.N.; Brown, J.R.; Taylor, C.M.; Huttenhower, C.; Langille, M.G.I. PICRUSt2: An Improved and Extensible Approach for Metagenome Inference. *bioRxiv* **2019**, 672295, doi:10.1101/672295.
105. Bolyen, E.; Rideout, J.R.; Dillon, M.R.; Bokulich, N.A.; Abnet, C.C.; Al-Ghalith, G.A.; Alexander, H.; Alm, E.J.; Arumugam, M.; Asnicar, F.; et al. Reproducible, Interactive, Scalable and Extensible Microbiome Data Science Using QIIME 2. *Nat Biotechnol* **2019**, *37*, 852–857, doi:10.1038/s41587-019-0209-9.
107. Stoddard, S.F.; Smith, B.J.; Hein, R.; Roller, B.R.K.; Schmidt, T.M. RrnDB: Improved Tools for Interpreting RRNA Gene Abundance in Bacteria and Archaea and a New Foundation for Future Development. *Nucleic Acids Res.* **2015**, *43*, D593–D598, doi:10.1093/nar/gku1201.
108. Quast, C.; Pruesse, E.; Yilmaz, P.; Gerken, J.; Schweer, T.; Yarza, P.; Peplies, J.; Glöckner, F.O. The SILVA Ribosomal RNA Gene Database Project: Improved Data Processing and Web-Based Tools. *Nucleic Acids Res.* **2012**, *41*, D590–D596, doi:10.1093/nar/gks1219.
109. Aßhauer, K.P.; Wemheuer, B.; Daniel, R.; Meinicke, P. Tax4Fun: Predicting Functional Profiles from Metagenomic 16S RRNA Data. *Bioinformatics* **2015**, *31*, 2882–2884, doi:10.1093/bioinformatics/btv287.

110. Edgar, R.C. UPARSE: Highly Accurate OTU Sequences from Microbial Amplicon Reads. *Nat. Methods* **2013**, *10*, 996–998, doi:10.1038/nmeth.2604.
111. Mori, H.; Maruyama, T.; Yano, M.; Yamada, T.; Kurokawa, K. VITCOMIC2: Visualization Tool for the Phylogenetic Composition of Microbial Communities Based on 16S RRNA Gene Amplicons and Metagenomic Shotgun Sequencing. *BMC Syst Biol* **2018**, *12*, 30, doi:10.1186/s12918-018-0545-2.
144. McDonald, D.; Price, M.N.; Goodrich, J.; Nawrocki, E.P.; DeSantis, T.Z.; Probst, A.; Andersen, G.L.; Knight, R.; Hugenholtz, P. An Improved Greengenes Taxonomy with Explicit Ranks for Ecological and Evolutionary Analyses of Bacteria and Archaea. *ISME J* **2012**, *6*, 610–618, doi:10.1038/ismej.2011.139.
145. Schloss, P.D.; Westcott, S.L.; Ryabin, T.; Hall, J.R.; Hartmann, M.; Hollister, E.B.; Lesniewski, R.A.; Oakley, B.B.; Parks, D.H.; Robinson, C.J.; et al. Introducing Mothur: Open-Source, Platform-Independent, Community-Supported Software for Describing and Comparing Microbial Communities. *Applied and Environmental Microbiology* **2009**, *75*, 7537–7541, doi:10.1128/AEM.01541-09.
146. Langille, M.G.I.; Zaneveld, J.; Caporaso, J.G.; McDonald, D.; Knights, D.; Reyes, J.A.; Clemente, J.C.; Burkepile, D.E.; Vega Thurber, R.L.; Knight, R.; et al. Predictive Functional Profiling of Microbial Communities Using 16S RRNA Marker Gene Sequences. *Nat. Biotechnol.* **2013**, *31*, 814–821, doi:10.1038/nbt.2676.
147. Cole, J.R.; Wang, Q.; Cardenas, E.; Fish, J.; Chai, B.; Farris, R.J.; Kulam-Syed-Mohideen, A.S.; McGarrell, D.M.; Marsh, T.; Garrity, G.M.; et al. The Ribosomal Database Project: Improved Alignments and New Tools for RRNA Analysis. *Nucleic Acids Res* **2009**, *37*, D141–D145, doi:10.1093/nar/gkn879.
148. Wang, Q.; Garrity, G.M.; Tiedje, J.M.; Cole, J.R. Naive Bayesian Classifier for Rapid Assignment of RRNA Sequences into the New Bacterial Taxonomy. *Applied and Environmental Microbiology* **2007**, *73*, 5261–5267, doi:10.1128/AEM.00062-07.
149. Yilmaz, P.; Parfrey, L.W.; Yarza, P.; Gerken, J.; Pruesse, E.; Quast, C.; Schweer, T.; Peplies, J.; Ludwig, W.; Glöckner, F.O. The SILVA and "All-Species Living Tree Project (LTP)" Taxonomic Frameworks. *Nucl. Acids Res.* **2014**, *42*, D643–D648, doi:10.1093/nar/gkt1209.
150. Caporaso, J.G.; Kuczynski, J.; Stombaugh, J.; Bittinger, K.; Bushman, F.D.; Costello, E.K.; Fierer, N.; Peña, A.G.; Goodrich, J.K.; Gordon, J.I.; et al. QIIME Allows Analysis of High-Throughput Community Sequencing Data. *Nat Methods* **2010**, *7*, 335–336, doi:10.1038/nmeth.f.303.
151. López-García, A.; Pineda-Quiroga, C.; Atxaerandio, R.; Pérez, A.; Hernández, I.; García-Rodríguez, A.; González-Recio, O. Comparison of Mothur and QIIME for the Analysis of Rumen Microbiota Composition Based on 16S RRNA Amplicon Sequences. *Front Microbiol* **2018**, *9*, 3010, doi:10.3389/fmicb.2018.03010.
152. Mothur Available online: https://mothur.org/ (accessed on 10 September 2019).
153. Balvočiūtė, M.; Huson, D.H. SILVA, RDP, Greengenes, NCBI and OTT — How Do These Taxonomies Compare? *BMC Genomics* **2017**, *18*, 114, doi:10.1186/s12864-017-3501-4.
154. Federhen, S. The NCBI Taxonomy Database. *Nucleic Acids Research* **2012**, *40*, D136–D143, doi:10.1093/nar/gkr1178.
155. Staley, C.; Gould, T.J.; Wang, P.; Phillips, J.; Cotner, J.B.; Sadowsky, M.J. Sediments and Soils Act as Reservoirs for Taxonomic and Functional Bacterial Diversity in the Upper Mississippi River. *Microb Ecol* **2016**, *71*, 814–824, doi:10.1007/s00248-016-0729-5.
156. Roller, B.R.K.; Stoddard, S.F.; Schmidt, T.M. Exploiting RRNA Operon Copy Number to Investigate Bacterial Reproductive Strategies. *Nature Microbiology* **2016**, *1*, 16160, doi:10.1038/nmicrobiol.2016.160.
